# The Dental Profession

**Published:** 1870-03

**Authors:** 


### Editorial.

### THE DENTAL PROFESSION.

A close examination and analysis of our profession will reveal the fact that the attention and efforts of a large proportion of those recognized by the public as Dentists are directed to the production of artificial dentures. With them, the preservation of the natural teeth, and their restoration to health, when diseased, are considerations of a secondary character. This is an exceedingly unfortunate state of affairs in our profession, and there is but little prospect of much immediate change. The facility with which a knowledge of the method of making artificial dentures was acquired was so great, upon the introduction of hard rubber, that perhaps thousands entered by this door who never could or would have come into the profession in any other way.

Quite a number have written to us that they are compelled to abandon the practice, because they can not comply with the demands of the Rubber Company; that is, if they can not use rubber, out of which to make artificial dentures, they can not do enough in any other particular of practice to warrant them in keeping open an office. Oh! that the Rubber Company, or some other influence had the power to close every such office in the country! Why is it that some good and influential practitioners, in our profession, will encourage and urge on such men? But perhaps there are some men of this class who should not be utterly condemned; men who have, in a mechanical way, made attainments sufficient to enable them to construct a passably good artificial denture, one that may be of service to the unfortunate. Now, if every such one will content himself with the occupancy of his true position, and not try to pass himself for a pound when he is but a shilling, he will find little or no hostility from any quarter, and especially if he will bear testimony within his sphere of action that there are pounds as well as shillings.

There seem to be some difficulties, however, at present, at least, inseparably connected with that class of the profession of whom we are speaking. In every community where they exist, and they are everywhere, a large majority of the people accept them as fair representatives of our profession, though they do not recognize them as having the attainments really possessed by the true Dentist, but the latter is regarded in the popular estimation as down upon the low plain of the former. In almost every community, high professional attainments and integrity, have this difficulty to contend with.

No Dentist receives that stimulus and encouragement from all his patients which will urge him forward in making the highest attainments, and in doing all it is possible for him to do for their best interests.

If he avails himself of the best facilities, in the way of expensive appliances and instruments, he is regarded as a spendthrift, or as getting up some clap-trap to get more money from his patients; and if he leaves his office for a time, to repair wasted energies, and by associative effort with his professional brethren, endeavors to increase his store of knowledge and ability to do his patients good, ten to one they will charge him with being away from his business, neglecting them, and spending money that he ought to save.

Now all this, to a large extent, is the result of the degraded level to which the whole profession has been dragged by the class of practitioners referred to in the beginning of this article.

Now what shall be the remedy for all this? We will suggest but one practical road, and that is, to make the line of distinction between the Dentist and the mere mechanical tooth-maker as deep and broad as possible. By this we do not mean to degrade the latter an iota, but to elevate him many fold, if possible; (there are very many cases we wot of in which this would be quite admissible), but let his sphere be distinctly understood, in the profession and with the public; and that of the true Dental physician and surgeon as distinctly defined and understood.

The manufacturers of artificial dentures should no more extract teeth, or perform any surgical operations upon the mouth than the maker of artificial limbs should perform operations upon the natural ones.

In the manufacture of artificial dentures we would place no restrictions; let all engage in it who can or wish to; but it should be far otherwise with those who propose to treat and operate upon the living organs. They should have that knowledge that would enable them always to avoid mistakes and to secure the greatest possible good to those under their care. T.

